# DNA methylation of the long intergenic noncoding RNA 299 gene in triple-negative breast cancer: results from a prospective study

**DOI:** 10.1038/s41598-020-68506-0

**Published:** 2020-07-16

**Authors:** Mehdi Manoochehri, Michael Jones, Katarzyna Tomczyk, Olivia Fletcher, Minouk J. Schoemaker, Anthony J. Swerdlow, Nasim Borhani, Ute Hamann

**Affiliations:** 1 Molecular Genetics of Breast Cancer, German Cancer Research Center (DKFZ), Heidelberg, Germany; 2 The Institute of Cancer Research, London, UK; 3 The Breast Cancer Now Toby Robins Research Centre, The Institute of Cancer Research, London, UK; 4 Division of Breast Cancer Research, The Institute of Cancer Research, London, UK

**Keywords:** Cancer, Genetics, Molecular biology, Biomarkers

## Abstract

Triple-negative breast cancer (TNBC) is an aggressive breast cancer subtype associated with a high rate of recurrence and poor prognosis. Recently we identified a hypermethylation in the long noncoding RNA 299 (*LINC00299*) gene in blood-derived DNA from TNBC patients compared with healthy controls implying that *LINC00299* hypermethylation may serve as a circulating biomarker for TNBC. In the present study, we investigated whether *LINC00299* methylation is associated with TNBC in a prospective nested breast cancer case–control study within the Generations Study. Methylation at cg06588802 in *LINC00299* was measured in 154 TNBC cases and 159 breast cancer-free matched controls using MethyLight droplet digital PCR. To assess the association between methylation level and TNBC risk, logistic regression was used to calculate odd ratios and 95% confidence intervals, adjusted for smoking status. We found no evidence for association between methylation levels and TNBC overall (*P* = 0.062). Subgroup analysis according to age at diagnosis and age at blood draw revealed increased methylation levels in TNBC cases compared with controls in the young age groups [age 26–52 (*P* = 0.0025) and age 22–46 (*P* = 0.001), respectively]. Our results suggest a potential association of *LINC00299* hypermethylation with TNBC in young women.

## Introduction

Triple-negative breast cancer (TNBC) is an aggressive breast cancer subtype accounting for 15% of breast cancer in women of Caucasian descent^1^. It is defined by lack of estrogen receptor (ER), progesterone receptor (PR), and human epidermal growth factor receptor 2 (HER2) expression. TNBC is associated with younger age at diagnosis, higher tumor grade, and advanced disease stage and is associated with an increased risk of recurrence and worse survival relative to other subtypes^2^. The absence of expression of the three receptors strongly reduces targeted treatment options and as such, there is an urgent need to identify novel targets for treatment^3,4^ or improve early detection.

Aberrant DNA methylation is reported in tumor tissue of many cancers including breast cancer^5,6^. Similar alterations are detectable in peripheral blood leukocyte (PBL) DNA from cancer patients implying that blood-based DNA methylation markers may be of clinical value for early detection and risk stratification^7,8^. In breast cancer, several global and gene-specific DNA methylation studies have been performed on PBL-derived DNA^9^. Global DNA methylation measures have yielded inconsistent findings^9,10^. There is evidence that local hypermethylation at the *BRCA1*^11,12^ gene promoter and hypermethylation at the *ATM* gene body^13,14^ in PBL DNA is more frequent in breast cancer cases compared with controls. Hypermethylation of the *BRCA1* promoter in PBL DNA was also associated with a greater risk of TNBC compared to other breast subtypes, indicating its application as a novel methylation biomarker of increased TNBC susceptibility^15^. In another study hypermethylation of *DOK7* in whole blood DNA was proposed as a powerful epigenetic blood-based biomarker for TNBC^16^.

Using a retrospective study design, we recently identified and validated a region within the long noncoding RNA 299 (*LINC00299*) gene that showed a higher methylation (3% in the discovery set and 2% in the validation set) in PBL DNA from TNBC patients compared with healthy controls^17^, suggesting that this may be a biomarker for TNBC. The hypermethylated region is located in a putative regulatory region of the *LINC00299* gene, the function of which is unknown.

In the present study, we tested whether *LINC00299* methylation level is associated with TNBC using a prospective study design. Methylation was measured in PBL DNA from 154 TNBC cases and 159 matched controls in a nested case–control study within the prospective Generations Study (GS) cohort using droplet digital PCR (ddPCR).

## Results

Methylation at cg06588802 in *LINC00299* was measured in peripheral blood DNA of 154 TNBC cases and 159 matched controls from the study. Selected characteristics of the study participants are shown in Table 1.

**Table 1:** Characteristics of triple negative breast cancer cases and controls [matched on year of study entry, age at entry, days blood was in the post before processing, and cancer-free years in the study (time at risk)].

Characteristic	Cases n (%)	Controls n (%)
Total^a^	154	159
Age at study entry [mean (range), years]	52.1 (24–80)	52.1 (22–81)
Year of study entry	2004–2010	2004–2009
Age at blood draw [mean (range), years]	52.1 (24–80)	52.2 (22–81)
Age at diagnosis date [mean (range), years]	56.7 (26–87)	–
Blood draw to diagnosis [mean (range), years]	4.6 (0–10)	–
**Days blood in post**		
0–1	116 (75.3)	120 (75.5)
2	11 (7.1)	11 (6.9)
3+	27 (17.5)	28 (17.6)
**Smoking status**		
Never	94 (61.0)	94 (59.1)
Ex-smoker	51 (33.1)	53 (33.3)
Current	9 (5.8)	12 (7.6)

*TNBC* triple negative breast cancer.

^a^Including twelve participants with prior cancer (that is not breast cancer).

Mean methylation levels were higher in TNBC cases compared with controls. However, this difference did not reach statistical significance (*P* = 0.062). In analyses stratified by age, women in the lowest tertile of age at diagnosis (26–52) and age at blood draw (22–46) had statistically significant higher mean methylation levels in cases compared with controls [*P* = 0.0025 and *P* = 0.0010, respectively; (post-hoc power > 90% in both instances); Table 2]. No significant differences between cases and controls were detected for women in the second and third age tertiles.

**Table 2:** Difference in methylation levels for triple negative breast cancer cases versus controls: matched analysis (analysis of variance), stratified by age (tertiles).

	Cases		Controls		Matched analysis^a^
Strata	n	Mean methylation level (SD)	n	Mean methylation level (SD)	*P* value
	154	0.430 (0.051)	159	0.420 (0.051)	0.062
**Age at diagnosis**					
26–52	50	0.453 (0.036)	51	0.423 (0.043)	0.0025
53–62	53	0.433 (0.044)	56	0.429 (0.053)	0.76
63+	51	0.404 (0.059)	52	0.405 (0.056)	0.94
Heterogeneity (*df* = 3)^b^					0.025
**Age at blood draw**					
22–46	48	0.454 (0.036)	52	0.427 (0.042)	0.0010
47–58	59	0.429 (0.049)	58	0.422 (0.055)	0.46
59+	47	0.406 (0.056)	49	0.410 (0.057)	0.54
Heterogeneity (*df* = 3)^b^					0.0079

*SD* standard deviation, *df* degrees of freedom.

^a^Controls matched on year of study entry, age at study entry, days blood in post before processing, and cancer-free years (time at risk).

^b^Heterogeneity test: tests if the difference between cases and controls varies by age.

Analysis by quartile revealed no association between methylation levels at cg0658802 in *LINC00299* and TNBC risk, adjusted for smoking status (Table 3). Similar results were obtained after exclusion of twelve study participants with prior cancer and their matched case or control (Q2: OR 0.64, 95% CI 0.29–1.41; Q3: OR 1.58, 95% CI 0.78–3.20; Q4: OR 1.08, 95% CI 0.51–2.31).

**Table 3:** Odds ratio of triple negative breast cancer in relation to methylation levels at cg06588802 in *LINC00299*, all ages.

Methylation level						
Quartile (Q)	Mean	Cases n	Controls n	OR^a^	95% CI	*P* value
Total^b^		154	159			
Q1 (0.289–0.388)	0.354	34	41	1.00	Baseline	
Q2 (0.389–0.420)	0.406	26	39	0.80	0.37–1.71	0.56
Q3 (0.421–0.461)	0.441	51	40	1.51	0.77–2.98	0.23
Q4 (0.462–0.558)	0.486	43	39	1.27	0.61–2.63	0.53
Trend (across quartiles)						0.14
Trend (across methylation ratio)						0.082

*OR* odds ratio, *CI* confidence interval.

^a^Conditional matched analysis [year of entry to study, age at entry, days blood in post before processing, and cancer-free years (time at risk)], adjusted for smoking status.

^b^Including twelve participants with prior cancer (that is not breast cancer).

## Discussion

This is the first study to investigate an association between TNBC and gene-specific methylation in prediagnostic blood samples of TNBC cases and age-matched controls using ddPCR as a highly quantitative method. We found no evidence for association of *LINC00299* methylation levels with TNBC overall. However, in analysis stratified by age at diagnosis and age at blood draw, higher methylation levels were observed in the youngest age subgroup of TNBC cases compared with controls, but not in the older age subgroups. These findings imply that *LINC00299* methylation level may be useful as a biomarker for TNBC in young women, which has not been examined previously. The different results obtained in the age subgroups might be explained by differences in molecular features of TNBC between younger and older women. One study for example showed that women younger than 50 years at the time of diagnosis had a higher prevalence of TNBC of the basal-like molecular subtype and a lower prevalence of the HER2-enriched subtype compared with those who were older at the time of diagnosis (≥ 65 years)^18^. However, we did not have this level of molecular subtyping in our cases to be able to examine this further. Also younger women were more likely to develop TNBCs with poor prognostic parameters, such as higher histological grade, higher number of positive lymph nodes, larger tumor size, higher proliferation rate, and higher TNM stage^2^. It is also reported that *BRCA1* promoter methylation in peripheral blood increases the risk of having early onset breast cancer^19^. Furthermore, it was reported that younger TNBC patients (< 40 years) had a worse survival than their older (> 50 years) counterparts^20,21^.

A statistically significant methylation difference between TNBC cases and controls was reported in our initial study^17^. In the present study, however, this difference did not reach statistical significance. The main difference between the studies is that in the present study blood samples were taken from women before the diagnosis of TNBC, excluding the possibility that methylation variability in PBL DNA was modified by the presence of clinical cancer or treatment of these patients. In the previous study, blood samples were drawn at the time of TNBC diagnosis and tumors can directly influence the host immune system by releasing factors that modulate functions of leukocytes or induce apoptosis of these cells^22^. This may lead to changes in PBL DNA methylation levels. Other explanations may be differences in study size, population, and matching criteria (matched on age at study entry ± 5-years, year of study entry, days blood in post before processing, and cancer-free years (time at risk) versus matched on age at diagnosis for cases and interview for controls ± 1 year).

The CpG site cg06588802 is located at the chromosomal region 2p25.1 within the *LINC00299* gene. LncRNAs have recently emerged as important regulators of gene expression in various cell types. They control the development and function of specific immune cells through a variety of mechanisms^23^. The hypermethylated region in the *LINC00299* gene is evolutionarily conserved and overlaps with several enhancer regions suggesting its possible regulatory functions. Data of the three-dimensional chromatin structure showed physical interactions between the genomic region of *LINC00299* and the *ID2* gene promoter, a protein with important immune functions^24^. This suggests a potential function of *LINC00299* in the regulation of specific immune cells, which needs to be elucidated in functional studies. Further, based on expression data from The Cancer Genome Atlas, ER- and PR-negative breast cancer patients with high *LINC00299* expression had a better survival than those with low expression^17^.

In conclusion, though no association between *LINC00299* methylation and TNBC overall was observed, our findings suggest that *LINC00299* hypermethylation in prediagnostic PBL DNA may be associated with TNBC in young women. If replicated in larger studies, *LINC00299* hypermethylation may be of clinical value as a biomarker for early-detection of TNBC in young women.

## Methods

### Study population

Study participants were selected from the GS, a long-term prospective breast cancer cohort study focused on potential etiological factors for breast cancer in women in the UK, with blood samples collected at recruitment^25^. The study has been approved, under the procedures for national medical research studies, by the South-East Multi-Centre Research Ethics Committee.

The present study selected 161 TNBC cases and matched controls of Caucasian ethnicity for methylation assay. Cases were women who were diagnosed with a first triple-negative invasive or in situ primary breast cancer after study entry. Controls were women with no diagnosis of breast cancer. Individual controls were selected for each case, matched on age (5-year categories), year of study entry (≤ 2005, 2006, 2007, ≥ 2008), the number of days the blood had been in the post (0–1, 2, ≥ 3), and cancer-free years (time at risk). After updating data files prior to statistical analysis, four cases were subsequently found to have provided blood samples after diagnosis of TNBC and therefore were excluded from the analysis. Further, three cases and two controls had missing methylation data. Hence, nine women (two cases/seven controls) were no longer in a matched pair; these nine women were re-allocated to a matched set with the same age, study entry year, days blood in post grouping, but different cancer free time (thus allowing the inclusion of these women in the matched analysis). In total, the study included 154 cases (diagnosed with a first triple-negative invasive (n = 149) or in situ (n = 5) primary breast cancer after study entry) and 159 controls.

### Methylation analysis

DNA samples were extracted from buffy coats using DNA Blood Mini Kits (Qiagen, Hilden, Germany). Two hundred ng of DNA were bisulfite-converted using EpiTect Fast 96 Bisulfite Conversion Kit according to the manufacturer's protocol (Qiagen). DNA methylation analysis at cg06588802 in *LINC00299* was performed using MethyLight droplet digital PCR (ddPCR) as previously described^17^. In brief, methylation ratios were defined by locus-specific primers using TaqMan assays. The C-LESS-C1 assay was used as internal control for normalization. Each reaction was performed in a final volume of 20 μl containing 10 μl ddPCR Supermix for Probes (No dUTP) (Bio-Rad, Hercules, CA, USA), 900 nM forward and reverse primers, 250 nM probe, 2 µl bisulfite converted DNA template, and 6 µl nuclease-free double distilled H_2_O. All ddPCR steps were performed according to the manufacturer’s protocols (Bio-Rad). Cycling was at 95 °C for 10 min, followed by 40 cycles of 94 °C for 30 s and 59 °C for 60 s, and a final step at 98 °C for 10 min. To ensure equal conditions for cases and controls during all ddPCR steps, case–control pairs were allocated on the same cartridge of the assay to minimize batch effects. Duplicates and fully methylated and unmethylated controls were used on each ddPCR plate for quality control.

### Statistical analysis

Analysis of variance was used to assess the difference in methylation levels between TNBC cases and matched controls (i.e. with implicit adjustment for the matching factors).

Stratified analysis (tertiles) was performed by age at diagnosis (for controls this was the matched case’s age at diagnosis) and age at blood draw.

For the association between methylation level and TNBC risk, conditional logistic regression was used to calculate odd ratios (ORs) and 95% confidence intervals (CIs). We adjusted for smoking status (at recruitment: never, ex-smoker, current) as *LINC00299* methylation level is changed in response to cigarette smoking^26^.

All *P* values were two-sided, with a *P* value of 0.05 considered statistically significant. Stata/IC version 14.2 was used for all analyses^27^.

### Ethical approval

All procedures performed in studies involving human participants were in accordance with the ethical standards of the institutional and/or national research committee and with the 1964 Helsinki declaration and its later amendments or comparable ethical standards. The study has been approved by the South-East Multi-Centre Research Ethics Committee.

### Informed consent

Informed consent was obtained from all individual participants included in the study.

## Data Availability

Data sharing not applicable to this article as no datasets were generated or analyzed during the current study.

## References

[1] Plasilova ML (2016). Features of triple-negative breast cancer: analysis of 38,813 cases from the national cancer database. Medicine (Baltimore).

[2] Zhu W, Perez EA, Hong R, Li Q, Xu B (2015). Age-related disparity in immediate prognosis of patients with triple-negative breast cancer: a population-based study from SEER cancer registries. PLoS ONE.

[3] Li X (2017). Triple-negative breast cancer has worse overall survival and cause-specific survival than non-triple-negative breast cancer. Breast Cancer Res. Treat..

[4] Bianchini G, Balko JM, Mayer IA, Sanders ME, Gianni L (2016). Triple-negative breast cancer: challenges and opportunities of a heterogeneous disease. Nat. Rev. Clin. Oncol..

[5] Saghafinia S, Mina M, Riggi N, Hanahan D, Ciriello G (2018). Pan-cancer landscape of aberrant DNA methylation across human tumors. Cell Rep..

[6] Amini M (2019). GHSR DNA hypermethylation is a new epigenetic biomarker for gastric adenocarcinoma and beyond. J. Cell. Physiol..

[7] Jung K, Fleischhacker M, Rabien A (2010). Cell-free DNA in the blood as a solid tumor biomarker—a critical appraisal of the literature. Clin. Chim. Acta.

[8] Li L (2012). DNA methylation in peripheral blood: a potential biomarker for cancer molecular epidemiology. J. Epidemiol..

[9] Tang Q, Cheng J, Cao X, Surowy H, Burwinkel B (2016). Blood-based DNA methylation as biomarker for breast cancer: a systematic review. Clin. Epigenet..

[10] Bodelon C (2019). Blood DNA methylation and breast cancer risk: a meta-analysis of four prospective cohort studies. Breast Cancer Res..

[11] Iwamoto T, Yamamoto N, Taguchi T, Tamaki Y, Noguchi S (2011). BRCA1 promoter methylation in peripheral blood cells is associated with increased risk of breast cancer with BRCA1 promoter methylation. Breast Cancer Res. Treat..

[12] Snell C (2008). BRCA1 promoter methylation in peripheral blood DNA of mutation negative familial breast cancer patients with a BRCA1 tumour phenotype. Breast Cancer Res..

[13] Brennan K (2012). Intragenic ATM methylation in peripheral blood DNA as a biomarker of breast cancer risk. Cancer Res..

[14] Flanagan JM (2009). Gene-body hypermethylation of ATM in peripheral blood DNA of bilateral breast cancer patients. Hum. Mol. Genet..

[15] Prajzendanc K (2019). BRCA1 promoter methylation in peripheral blood is associated with the risk of triple-negative breast cancer. Int. J. Cancer.

[16] Heyn H (2013). DNA methylation profiling in breast cancer discordant identical twins identifies DOK7 as novel epigenetic biomarker. Carcinogenesis.

[17] Bermejo JL (2019). Long intergenic noncoding RNA 299 methylation in peripheral blood is a biomarker for triple-negative breast cancer. Epigenomics.

[18] Gulbahce HE (2018). Differences in molecular features of triple-negative breast cancers based on the age at diagnosis. Cancer.

[19] Wong EM (2011). Constitutional methylation of the BRCA1 promoter is specifically associated with BRCA1 mutation-associated pathology in early-onset breast cancer. Cancer Prev. Res. (Phila).

[20] Liedtke C (2015). The prognostic impact of age in different molecular subtypes of breast cancer. Breast Cancer Res. Treat..

[21] Dai D (2019). The prognostic impact of age in different molecular subtypes of breast cancer: a population-based study. PeerJ.

[22] Whiteside TL (2006). Immune suppression in cancer: effects on immune cells, mechanisms and future therapeutic intervention. Semin. Cancer Biol..

[23] Mowel WK, Kotzin JJ, McCright SJ, Neal VD, Henao-Mejia J (2018). Control of immune cell homeostasis and function by lncRNAs. Trends Immunol..

[24] Yang CY (2011). The transcriptional regulators Id2 and Id3 control the formation of distinct memory CD8^+^ T cell subsets. Nat. Immunol..

[25] Swerdlow AJ (2011). The Breakthrough Generations Study: design of a long-term UK cohort study to investigate breast cancer aetiology. Br. J. Cancer.

[26] Tsaprouni LG (2014). Cigarette smoking reduces DNA methylation levels at multiple genomic loci but the effect is partially reversible upon cessation. Epigenetics.

[27] StataCorp. (2015). Stata Statistical Software: Release 14.

